# How does visual language affect crossmodal plasticity and cochlear implant success?

**DOI:** 10.1016/j.neubiorev.2013.08.011

**Published:** 2013-12

**Authors:** C.R. Lyness, B. Woll, R. Campbell, V. Cardin

**Affiliations:** a Cognitive, Perceptual and Brain Sciences, 26 Bedford Way, University College London, London WC1H 0AP, UK; b Deafness, Cognition and Language Research Centre, 49 Gordon Square, University College London, London WC1H 0PD, UK; c Linnaeus Centre HEAD, Swedish Institute for Disability Research, Department of Behavioural Sciences and Learning, Linköping University, Sweden

**Keywords:** Cochlear implant, Deafness, Functional decoupling, Crossmodal reorganisation, Delayed/insecure language acquisition

## Abstract

Cochlear implants (CI) are the most successful intervention for ameliorating hearing loss in severely or profoundly deaf children. Despite this, educational performance in children with CI continues to lag behind their hearing peers. From animal models and human neuroimaging studies it has been proposed the integrative functions of auditory cortex are compromised by crossmodal plasticity. This has been argued to result partly from the use of a visual language. Here we argue that ‘cochlear implant sensitive periods’ comprise both auditory and language sensitive periods, and thus cannot be fully described with animal models. Despite prevailing assumptions, there is no evidence to link the use of a visual language to poorer CI outcome. Crossmodal reorganisation of auditory cortex occurs regardless of compensatory strategies, such as sign language, used by the deaf person. In contrast, language deprivation during early sensitive periods has been repeatedly linked to poor language outcomes. Language sensitive periods have largely been ignored when considering variation in CI outcome, leading to ill-founded recommendations concerning visual language in CI habilitation.

## Highlights

• Children with CI perform poorer than their hearing peers in educational settings.
• CI sensitive periods are influenced by language and auditory deprivation.
• Reorganisation of auditory cortex is an inevitable consequence of deafness.
• Visual language does not influence crossmodal plasticity.
• Early language deprivation has deleterious consequences for language learning.

## 1 Introduction

The advent of paediatric cochlear implants (CIs) has been a significant achievement in restoring hearing (Archbold and Mayer, 2012). With a CI, the 5000 inner hair cells of the human cochlea are replaced with up to 22 electrodes which directly stimulate the remaining auditory nerve fibres. This is not intended to replicate the auditory signal, but crudely simulates the main coding principles of the cochlea (Wilson et al., 2011). For the 1 in 1000 children in the UK who are severely or profoundly deaf by their third birthday (over 90% of whom are the children of hearing parents), cochlear implantation is recommended by clinicians in the majority of cases. This has resulted in a dramatic increase in the uptake of CIs in the past 10 years.

Children who receive implants in early childhood (<3 years) develop speech processing abilities, often far in advance of those predicted for a deaf child without a CI (Stacey et al., 2006). As age and duration of deafness increase, the positive effects of CIs become less predictable, although they can still be extremely effective in some cases (Markman et al., 2011). Extensive habilitation is required in order to achieve speech production and comprehension skills comparable to those of a hearing child. Even with such interventions, group studies suggest that the long-term prognosis for the child with CI does not always bring her within close range of the child with normal hearing (Venail et al., 2010; Geers et al., 2011). A large sample of US teenagers implanted with CI between 2 and 5 years was investigated in elementary school (CI-E tests) and again as teenagers (CI-HS tests) by Geers and colleagues. By their teenage years, nearly 30% of students were not within one standard deviation of hearing children on tests of simple verbal reasoning such as WISC III, and over half demonstrated a significant gap between their verbal intelligence quotient (VIQ) and their performance intelligence quotient (PIQ) (Geers and Sedey, 2011). Nearly 20% of students with CI made minimal progress in reading skills between CI-E and CI-HS testing sessions, and written expression remained a problem for the majority of CI students, with only 38% scoring within one standard deviation of the hearing students (Geers and Hayes, 2011). It is evident that a CI does not simply transform a deaf child into a hearing child, and a greater understanding of the reasons for these differences in CI outcome is needed if such difficulties are to be overcome. The aim of this article is to review the evidence on the effect of visual languages (Table 1) on neural function in deaf people, and their relation to CI success. We propose that auditory deprivation and delayed language acquisition have interacting effects. Until now, these effects have been confounded. Most researchers have implied that early experience with a visual language impacts negatively on CI outcome. However, these studies have failed to account for the level of (visual) language acquisition in the pre-implant child. We propose that this has led to a misleading perspective in relation to habilitation and intervention, and unjustified recommendations in relation to the use of visual language for deaf children with CIs.

## 2 Animal models of auditory cortical sensitive periods

Neural development is the result of a dynamic interplay between a genetically specified developmental trajectory and extrinsic environmental factors. A ‘sensitive period’ is a period of time during which the development of a particular brain function is very sensitive to external input (Knudsen, 2004). Sensitive periods have been shown for a variety of brain functions, including language, audition and vision. Deprivation of external input (or aberrant input) during a sensitive period will prevent typical development of neural circuitry for the particular function. When environmental input is restored after deprivation during the sensitive period, this alone will not normalise the affected brain circuitry (Hubel and Wiesel, 1977; Hensch, 2004; Knudsen, 2004; Hensch, 2005).

Animal models of CI have been developed to explore the neurophysiology of auditory deprivation, and what is often called the ‘cochlear implant sensitive period’ (Kral and Sharma, 2012). In this paradigm, deaf animals will be implanted after a certain period of time, and the effect of electrical stimulation on the neural circuitry for hearing examined. There is abundant evidence that early auditory input is a necessary pre-requisite for typical development of auditory cortex. If a congenitally deaf cat receives a CI before it is 3 months old, 5 months of electrical stimulation of the auditory nerve will restore local field potentials (LFPs) in auditory cortex to a level comparable to hearing cats (Kral et al., 2002). However, if cats are implanted after 6 months of age, no amount of electrical stimulation will normalise their LFPs (Kral et al., 2002). Further research has been completed on aberrant properties of auditory cortex, which do not normalise following electrical stimulation of the auditory nerve subsequent to the sensitive period (Kral et al., 2006a). In a comprehensive paper on the effect of congenital deafness on the cortical representation of interaural time differences, the amount of auditory cortex which responds to electrical stimulation of the auditory nerve was shown to be reduced (Tillein et al., 2010). This is likely to affect the absolute amount of information auditory cortex is capable of representing. Further, the maximum rate of firing for spike trains in auditory cortex was also reduced, which has consequences for representing stimuli with dynamically changing sound intensity (Tillein et al., 2010). The auditory cortex of congenitally deaf animals who receive CIs possesses a rudimentary capability to represent interaural time differences, which has been argued to be mediated by subcortical structures (Tillein et al., 2010), that develop before hearing onset (Heid et al., 1997). Cortical cochleotopy, which encodes place information from the cochlea, is also reduced in animals that have been deaf for an extended period of time (Raggio and Schreiner, 1999). Unlike many other properties of the auditory system, cochleotopy can be largely restored in neonatally deafened cats following chronic electric stimulation with a cochlear implant (Fallon et al., 2009).

Animal models of auditory deprivation have clarified many of the neurophysiological underpinnings of these functional deficits. Typical development of auditory cortex is characterised by an early period of synaptogenesis, and subsequent pruning to remove non-functional circuitry (Huttenlocher and Dabholkar, 1997). However, in congenitally deaf cats, the process of synaptogenesis is increased and delayed (Kral et al., 2005). Without environmental input to shape functional circuitry, pruning mechanisms are indiscriminate (Kral et al., 2005). Despite this, a level of residual plasticity remains (Kral et al., 2002; Schramm et al., 2002; Sharma et al., 2007).

This residual plasticity is, however, thought to be undermined by the process of ‘functional decoupling’ whereby primary auditory cortex is no longer capable of being modulated by higher auditory fields (Kral and Sharma, 2012). Proper function of auditory cortex is predicated on the basis that it is a densely reciprocally interconnected system, which enables consistent top-down and bottom-up comparisons of information (Kral and Sharma, 2012). Electrophysiological recordings and histological analyses display abnormalities in infragranular cortical layers, the posited neural locus of this integrative activity (Kral and Sharma, 2012). The partial decoupling of primary auditory cortex from modulation by higher order auditory fields has been proposed to contribute to the closure of the auditory sensitive periods (Kral et al., 2006b; Kral, 2007). It has been claimed that crossmodal reorganisation of visual and somatosensory processing to auditory cortex may impede the top-down modulation of primary auditory cortex by higher auditory fields (Kral et al., 2006b; Kral, 2007).

## 3 Sensitive periods for cochlear implantation in children: an interaction between language and auditory sensitive periods

There is no doubt that animal models provide detailed information on the neurophysiological sequelae of congenital and neonatal deafness. However, these ultimately fall short of providing a satisfactory model for paediatric CI. Their primary purpose is to provide a physiological basis for understanding how early hearing loss impacts complex auditory function - of which heard speech is the prime example in humans. However, heard speech is not simply an auditory/acoustic function; it also reflects linguistic development: language cannot be considered with an animal model. We propose the cochlear implant sensitive period should be considered to encompass both a sensitive period for auditory processing and a sensitive period for language processing.

Language acquisition starts in the womb (DeCasper and Spence, 1986; Moon et al., 1993), and sensitive periods for different heard language skills have been described, many of which occur during the first year of life (Kuhl, 2004). In deaf children raised in deaf families and exposed to a sign language as a first language, the developmental pattern of language acquisition follows the same time-course as that for spoken-and-heard language, with similar milestones, patterns of mastery, and adult language skills (Meadow-Orlans et al., 2004) (see Box 1). Similarly, deaf children raised in a cued-speech environment (see Table 1) from their earliest days can reach levels of mastery of spoken language within range of their hearing peers, when tested at school-age (LaSasso and Crain, 2010; Leybaert and LaSasso, 2010). If congenitally or early deaf children are not exposed to a visual language early in life, it is likely that these children will miss part of the sensitive period for language learning, which may contribute to poor language outcomes in spite of residual auditory plasticity.

Crossmodal reorganisation, implicating visual ‘takeover’ of the auditory modality, has been argued to contribute to the closing of the cochlear implant sensitive period (Kral and Sharma, 2012), as late implanted children tend to show poorer speech outcomes than one would expect given the observed residual plasticity in the auditory cortex of deaf animals. Arguments that visual takeover is linked to poor CI outcome are of paramount importance, as vision is the major modality through which deaf children can access language. Exposure to sign language has been linked to maladaptive crossmodal plasticity, forever compromising the ability of auditory cortex to process spoken language (Nishimura et al., 1999; Lee et al., 2001; Giraud and Lee, 2007). Experience with speech-reading prior to CI has been argued to disrupt crossmodal integration of auditory and visual information, biasing it in favour of visual information (Hirano et al., 2000). In some implantation programmes, sign language and speech reading are contra-indicated prior to CI for these reasons, and prevailing habilitation strategies for CI focus on training the auditory modality at the direct expense of the visual (Chan et al., 2000; Hogan et al., 2008; Yoshida et al., 2008; Ingvalson and Wong, 2013). The two aims of keeping the auditory cortex ‘pure’ for subsequent implantation by avoiding visual language, and delivering visual language within the sensitive period, are antagonistic. Here we question the assumption that visual language causes maladaptive plasticity in auditory cortex, impacting negatively upon the efficacy of CI.

## 4 Neural predictors of CI success

Pre-implant demographic characteristics, results from psychological testing, and patterns of neural metabolism have all been studied in terms of their relationship with post-implant outcomes in order to predict suitability for CI. It is widely accepted that the most important determinant of CI success is age at implant, with CI prior to 3.5 years of age associated with better outcomes, whereas the likelihood of achieving a good outcome when implanted later is drastically reduced (Sharma et al., 2005; Dorman et al., 2007; Geers et al., 2011). Additionally, some studies have proposed a link between poor speech outcomes and exposure to a visual language (Hirano et al., 2000; Lee et al., 2001; Doucet et al., 2006).

A methodological issue with studies on the neural correlates of CI success is that duration of deafness, biological age and experience with a visual language are highly correlated, making inferences about the separate effect of each of these problematic. An influential study which measured resting brain metabolism in prelingually deaf children prior to CI, associated hypometabolism in auditory cortex with good CI outcome, as assessed by auditory speech skill (Lee et al., 2001). The authors concluded that when the metabolism of auditory cortex of deaf children was at levels comparable to those of hearing adults, it was incapable of processing auditory signals owing to usurping of its function by crossmodal plasticity, accounting for poorer CI outcome (Lee et al., 2001). The authors posit that sign language is an example of a cognitive process which may contribute to this maladaptive crossmodal plasticity of auditory cortex. However, biological age, duration of deafness, speech perception and age at implantation were correlated in this study. Therefore, these results cannot determine if the increase in metabolism observed in the temporal cortex was a consequence of increased visual crossmodal plasticity due to prolonged periods of deafness (as the authors claim), or just the result of physiological maturation of the cortex, in which metabolic activity increases with biological age.

In fact, in a further study, Lee et al. (2005) showed that when biological age, duration of deafness and age at implantation were controlled or accounted for in the analysis, the best predictor of auditory speech skill was found to be pre-implant *hyper*metabolic activity in fronto-parietal regions (Lee et al., 2005, 2007). Patients with poor outcomes also showed increased activity in ventral visual areas (Lee et al., 2005). This study did not replicate the hypometabolism observed in auditory regions in Lee et al. (2001). Therefore, there is no convincing evidence that the extent of crossmodal takeover reported in auditory cortex is linked to poor CI outcome, independently of biological age and duration of deafness.

Increased metabolism in good CI performers relative to poor CI performers has been reported in inferior frontal gyrus, as well as angular gyrus, both of which have language functions (Giraud and Lee, 2007). The authors argue this is a result of ‘potential’ for these regions to represent abstract and language-based concepts (Giraud and Lee, 2007). As these regions are multimodal (MacSweeney et al., 2008a), language networks may already be active in children who go on to be good performers with CI. Additionally, these regions have been linked to executive function skills, including working memory and attention, and therefore could also contribute to the differences observed between poor and good performers with CI (Geers and Sedey, 2011; Colom et al., 2013; Ingvalson and Wong, 2013).

Increased metabolism in the ventral visual pathway was linked to visual takeover of auditory and language processing circuits, and ultimately poor CI outcome (Giraud et al., 2011). However, there is no explanation of why visual ventral stream processing was detrimental whereas dorsal stream processing within parietal cortex was beneficial (Giraud et al., 2011). This is particularly counter-intuitive, as sign language has been shown to result in reorganisation of visual motion processing, reversing typical biases in hearing non-signers for improved motion velocity thresholds in the left over the right visual field (Brozinsky and Bavelier, 2001, Brozinsky and Bavelier, 2004).

To test the role of crossmodal plasticity in CI outcome, scalp-based event related potentials (ERPs) were measured in response to abstract visual patterns that had previously been shown to drive the ventral visual processing stream (Doucet et al., 2006). In good speech perceivers (tested post CI), ERPs recorded posteriorly over visual cortices were greater than in poor speech perceivers and control participants, whereas in participants with poor speech comprehension, ERPs were greater and extended more anteriorly over temporal regions, in comparison to both good speech perceivers and control participants with normal hearing (Doucet et al., 2006). However, owing to the problem of inferring cortical activation from scalp-based recordings (the inverse problem), this does not necessarily correspond to the source of neural activity being in the visual cortex for good speech perceivers and in the auditory cortex for poor speech perceivers. Poor performers were also sign language users and were unable to communicate using speech. The authors concluded that CI is successful when there is *intra*modal plasticity in which the visual signal is used to support the degraded auditory signal (for example, lip reading in audiovisual speech), whereas *cross*modal plasticity whereby the auditory cortex comes to process visual stimuli, such as sign language, is maladaptive plasticity from the perspective of CI (Doucet et al., 2006). However, within the tested groups, six out of seven good performers were post-lingually deafened, whereas four out of six poor performers were prelingually deafened (Doucet et al., 2006). Since the authors do not report parental (sign) language status for these participants (and since fewer than 10% of deaf people have deaf parents) it seems likely that the prelingually deafened participants, while fluent signers as adults, had nevertheless not reached language processing skills equivalent to those native signers or hearing people using a spoken language (see Box 1). Their (sign) language skills would have most likely been acquired out of the optimal sensitive period for language (so-called ‘late L1 signers’; see Mayberry et al., 2002, Box 1). Therefore, these findings could alternatively be explained by poor first language development, rather than maladaptive plasticity which resulted from sign language use.

Some studies of post-CI auditory speech proficiency have assessed whether pre-CI exposure to seen speech interferes with the ability of the auditory cortex to support perception of heard speech. Using PET, Hirano et al. (2000) compared the regional cerebral blood flow (rCBF) of 12 deaf participants (6 prelingually deafened and 6 post-lingually deafened) and 12 hearing controls whilst at rest, and whilst listening to speech. All prelingually deafened participants received their CI after the age of 8. The authors found that at rest the prelingually deafened group had higher cerebral metabolism in secondary auditory areas in comparison to post-lingually deafened participants and the hearing control groups. However, this pattern of results reversed when listening to speech, and the prelingually deafened group had decreased activation relative to the post-lingually deafened group and hearing controls. These findings speak to the importance of early CI implantation for typical auditory cortex function, which we do not dispute. To address the question of whether visual language processing comes to occupy secondary auditory cortex, the authors additionally completed a follow up study with 3 of the prelingually deafened participants. For 2 participants with previous speech reading skills, but whose speech recognition was not subsequently improved by CI, activation was reported in posterior superior temporal sulcus (a higher order auditory area) during speechreading, but not during listening to verbal speech (Hirano et al., 2000). In contrast, the single participant who did not have any previous speechreading skills, but did have improved speech recognition post CI, activated superior temporal sulcus during listening, but not while speechreading. The authors concluded that developing speechreading skills prior to implant had a negative impact on CI efficacy. However, there are reasons to be sceptical: a sample size of three, the lack of comparison between participants with similar pre-CI speechreading experience but different levels of speech recognition after CI, and few details concerning the language background of the implantees, suggest that these findings may not generalise. Moreover, when auditory and visual information were presented together (as is the case in natural speech), participants with previous experience of speechreading out-performed those without previous experience.

To date, no study of neural function has systematically measured, prior to implantation, proficiency in visual language (either sign language or speechreading) in relation to outcomes following CI. A retrospective study has compared implanted children who had either deaf parents (and so were native sign language users) or hearing parents, at different stages post-implant, including immediately after implant, and 3, 6 and 12 months afterwards (Hassanzadeh, 2012). The deaf native signing group outperformed the deaf children with hearing parents on measures of speech perception (Auditory Perception Test), speech production (Speech Intelligibility Rating Scale) and language development (Speech Imitation Test) (Hassanzadeh, 2012). Though larger participant numbers and a prospective design would enhance confidence in these findings, they suggest researchers and clinicians alike need to take seriously the role of language development prior to implant in explaining variance in CI outcome. This is expanded upon below.

As is typical when working with special populations, group numbers are small and variance between participants is vast. As such, it is hazardous to make firm conclusions and generalisations about the role of visual language in CI success, particularly when experience with visual language tends to be correlated with duration of deafness and delayed language acquisition. Equally troublesome are reverse inferences about resting state brain activation patterns, particularly when behavioural data is absent. It therefore seems to us that there are no persuasive grounds for the proposal that visual language exposure, by ‘hijacking’ auditory cortical regions, is a causal factor in poor CI outcome.

Several studies suggest the converse hypothesis. Pre-implant speechreading in prelingually deaf children is a good predictor of post-implant auditory speech processing abilities (Bergeson et al., 2005). Children in educational environments which place an emphasis on speech reading skills (oral communication) outperform in perceptual tasks of auditory, visual and audiovisual speech children from total communication environments (TC – see Table 1) (Bergeson et al., 2005). Over time, performance between these groups became comparable in the auditory alone and audiovisual conditions, though children from the total communication group continued to lag on the visual speech condition. The benefit speech reading confers was argued to be a heightened sensitivity to the correlations between lip patterns and speech sound, which ultimately facilitates the extraction of phonological information (Bergeson et al., 2005, 2010). Bergeson et al. (2005) found auditory speech perception skills improved in the 5 years following implant, which contradicts other studies which report performance with auditory stimuli was static in the 2–4 year follow up period after CI, against the backdrop of improved speechreading skills (Tyler et al., 1997). When children are implanted within the sensitive period for cochlear implant, linguistic progress was not simply associated with early implantation, but also with properties of the mother's language input, such as mean length of utterance and expansions (Markman et al., 2011; Nittrouer et al., 2012; Szagun and Stumper, 2012).

There is evidence to suggest that visual information is required to support the impoverished auditory signal in CI, as even post-lingually deafened implantees who have the benefit of previously acquired representations of speech still overwhelmingly depend on visual information for good CI performance (Rouger et al., 2007, 2008). Developing proficiency with CI has been linked to activation in early visual areas, which becomes increasingly specific over time, and with increased proficiency of CI use (Giraud et al., 2001a,b; Giraud and Truy, 2002). In contrast, activation in primary auditory cortex increased with duration of implant use, but did not become more stimulus specific (Giraud et al., 2001b). This pattern of findings has been interpreted as evidence for the auditory and visual modalities mutually reinforcing each other to process the speech signal as delivered by CI (Giraud et al., 2001a,b; Rouger et al., 2007).

In summary, the use of sign language cannot be empirically linked to poor CI outcome, since pre-implant sign language proficiency has never been measured. When deaf children learn sign language within the critical period for language acquisition from their deaf parents, they outperform deaf children from hearing parents who have limited exposure to sign language following CI on measures of *auditory* language skills (Hassanzadeh, 2012). Proficiency in speech reading has been repeatedly linked to good CI outcomes. Evidence to the contrary is beset with methodological concerns. This suggests that habilitation strategies which emphasise training the auditory modality at the expense of the visual modality should be reconsidered.

## 5 Crossmodal plasticity in auditory cortex and its relationship to CI success

Primate studies have explored the anatomical and functional organisation of auditory cortex directly by detailed histological and invasive techniques, offering a guide to the localisation and connectivity of auditory fields and streams within the human brain. While unable to provide direct information about language processing, they can offer insights into auditory speech processing, where segregated functional streams have been described to identify the circuitry involved in distinctive functional processes (Hickok and Poeppel, 2004; Rauschecker and Scott, 2009). Auditory cortex in humans occupies lateral and superior parts of the temporal lobe, and shows concentric organisation, with hierarchical connections between neighbouring regions (Galaburda and Sanides, 1980; Pandya, 1995; Hackett, 2008). The central core region, including primary auditory cortex (A1), is located deep within the supra temporal plane in Heschl's gyrus. While the exact extent and distribution of A1 (primary auditory cortex) in humans can vary between individuals, as can the topography of Heschl's gyrus itself (Rademacher et al., 2001), the first projections from the auditory subcortical relays to auditory cortex are in this region. A1 is encircled by secondary auditory cortices (A2) extending onto the upper surface of the superior temporal gyrus, which in turn is surrounded by the belt area, which is bordered laterally by a parabelt region. Belt and parabelt regions which were first described in animal (macaque) models, are generally considered to be analogous to auditory association areas as described in classical neuropsychological and aphasiological literature. In this paper we refer to these regions as secondary auditory areas. They are located along the length of the superior temporal sulcus, extending to the temporoparietal junction caudally, the temporal poles rostrally and the superior temporal sulcus inferiorly (Howard et al., 2000).

Imaging studies with CIs tend to be performed using PET, as the metallic components of these devices are contraindicated for both fMRI and E/MEG. However, fMRI and E/MEG provide data with superior spatial and temporal resolution, respectively. Thus, imaging studies with these modalities in congenitally deaf people who have not had a cochlear implant can be informative with regards to crossmodal reorganisation which occurs in the case of congenital deafness.

In fMRI studies, secondary auditory areas in deaf groups are activated by a wide range of visual stimuli, including sign language (Neville and Bavelier, 1998; Petitto et al., 2000; MacSweeney et al., 2002a, 2004; Corina et al., 2007; Capek et al., 2008; MacSweeney et al., 2008b; Emmorey et al., 2011; Cardin et al., 2013), biological motion including non-linguistic gesture (Allison et al., 2000; MacSweeney et al., 2004; Corina et al., 2007), and moving dots (Finney et al., 2001; Sadato et al., 2004; Fine et al., 2005). However, in a comprehensive review of crossmodal plasticity, substantial inter-individual variability between deaf participants has been reported in terms of the extent and location of visual processing in auditory cortex (Bavelier and Neville, 2002). Indeed, some studies do not find any visual crossmodal plasticity in these regions (Hickok et al., 1997). Secondary auditory areas have been hypothesised to be responsive to phonologically structured input (Petitto et al., 2000). However, British Sign Language (BSL) and Tic-Tac, an idiosyncratic manual-brachial gestural code used by UK race-course bookmakers, both elicited activation in secondary auditory cortex, despite the lack of linguistic structure in Tic-Tac (MacSweeney et al., 2004). This demonstrated that these regions process complex visual stimuli, independently of their linguistic content. Comparable activation was also found in the hearing participants. However, further studies have shown that the left superior temporal cortex and the anterior medial part of right superior temporal cortex of deaf individuals are preferentially responsive to sign language stimuli, over more general visual stimuli (Fine et al., 2005; MacSweeney et al., 2008a; Cardin et al., 2013). These regions are typically associated with speech processing in hearing individuals, and as such have been argued to be multimodal language regions (MacSweeney et al., 2008a).

Silent speechreading also activates lateral parts of the superior temporal plane in hearing adults, including lateral regions within Heschl's gyrus (Calvert et al., 1997, 2000; MacSweeney et al., 2002a; Calvert and Campbell, 2003; Reale et al., 2007; Capek et al., 2008). Furthermore, the more attentive (Pekkola et al., 2005) and skilled the speechreader (Capek et al., 2010), the greater the activation in these regions, demonstrating that this is behaviourally relevant. Contrasting audiovisual and (purely) auditory speech activation demonstrates that adding clear vision to audition can enhance activation in primary auditory areas (Calvert et al., 2000; Reale et al., 2007). Auditory imagery alone cannot explain these findings, as in prelingually deaf participants, seen speech generates extensive activation throughout auditory cortex (Capek et al., 2008, 2010). Research with typically hearing people suggests posterior superior temporal regions within secondary auditory cortex act as a dynamic ‘hub’ for such audiovisual integration (Calvert et al., 2000; Lee and Noppeney, 2011; McGettigan et al., 2012). fMRI studies in hearing adults who are proficient signers show that language processing regions, such as the left superior temporal cortex and inferior frontal gyrus, are also activated in response to sign language stimuli (Bavelier et al., 1998; MacSweeney et al., 2002b). The anterior temporal lobe has been suggested to be an amodal semantic knowledge hub (Patterson et al., 2007). Additionally, anterior ventral temporal cortex is activated in response to words, irrespective of auditory or visual presentation of these stimuli (Marinkovic et al., 2003; Chan et al., 2011). Thus, in hearing groups there is a consensus that regions in temporal cortex are engaged in modality independent processing of language. It is therefore not surprising these regions are engaged by deaf people processing sign language. These various studies all show visual language activation within auditory brain regions irrespective of hearing status, weakening the proposal that visual language causes crossmodal reorganisation of auditory areas in deaf people.

Crossmodal reorganisation of primary auditory cortex in deaf people is more contentious. Whether visual afferents become rewired into auditory cortex, resulting in involvement in early visual sensory processing, has been addressed by comparing signals from early (80–120 ms) and late (300–350 ms) windows for hearing and deaf people listening to speech and viewing sign language using MRI constrained magnetoencephalography (MEG) (Leonard et al., 2012). These windows were respectively argued to correspond to sensory processing and lexicosemantic processing of the stimuli (Leonard et al., 2012). Deaf participants did not have activation in primary or secondary auditory cortex during the early sensory processing window; however, both deaf and hearing individuals had activation in a frontotemporal network including superior temporal regions surrounding auditory cortex for the later lexicosemantic window (Leonard et al., 2012), suggesting that the responses to sign language stimuli observed in temporal cortices are related to language processing, regardless of modality, and not general visual processing. Activation in response to moving dot stimuli has been reported in primary auditory regions for deaf, but not for hearing people (Finney et al., 2001; Fine et al., 2005). However, this result comes from a study in which brain activity was averaged across participants. Primary auditory cortex is a small area, characterised by a high degree of anatomical variability (in terms of position and anatomical variants) (Hackett, 2008; Dick et al., 2012). Genuine activation emanating from this region could potentially be suppressed in group averaging procedures, or alternatively, activation from adjacent gyri could be smoothed into this area. Differences in white matter between deaf and hearing people in auditory cortex have been reported (Emmorey et al., 2003), which further increases the chances of group normalisation processes misrepresenting the location of activations, as deaf brains will have to be distorted to a greater extent during normalisation processes.

More recent fMRI studies that avoid smoothing and use anatomical definitions of primary auditory regions have shown that visual plasticity is at best slight in these regions, either when the visual stimulus is basic (flashing lights, Karns et al., 2012) or when it is complex (sign language, Cardin, 2013). Karns et al. (2012) found that visual stimulation resulted in differences between deaf and hearing participants only in the posterior region of the contralateral Heschl's gyrus. This difference was at least partially driven by a deactivation in the hearing group, consistent with previous literature in hearing individuals showing deactivation of unstimulated sensory cortices, or cortices which are unresponsive to the modality of the unattended stimuli (Laurienti et al., 2002; Johnson and Zatorre, 2005). Karns et al. (2012) do not report if the activation observed in the deaf group differed from the baseline value. Larger activations were observed in the deaf group in a bimodal condition (somatosensory and visual), in which participants attended to the visual stimuli. However, these bimodal activations did not differ from those obtained exclusively with somatosensory stimuli, which again suggests they were not driven by the presence of visual stimuli.

Cardin (2013) used more complex and dynamic visual stimulation (sign language actions) to investigate plasticity in A1. She reported differences between deaf and hearing individuals in subregions Te1.0 and Te1.2 of Heschl's gyrus, mainly driven by a deactivation in the hearing group rather than activation over baseline in the deaf group. However, Cardin (2013) did find a small, but significantly different from baseline, response to visual stimulation in Te1.2 specific to the deaf group. Also, Karns et al. (2012) found that activations in the rostro-lateral region of Heschl's gyrus (putatively Te1.2) correlated with the perceptual illusion of two visual flashes, when only one was presented, accompanied by two somatosensory flashes. These recent results suggest a small degree of visual crossmodal plasticity in the most lateral region of Heschl's gyrus (area Te1.2). However, this region is likely to be outside of primary sensory areas: it has been proposed to be an intermediate stage between core regions and higher processing areas (Morosan et al., 2001), and its cytoarchitectonic features do not correspond with the highly myelinated core region (Dick et al., 2012). Furthermore, the level of crossmodal plasticity was the same in deaf individuals who are native users of a sign language, and in those who communicate orally and do not use a sign language, suggesting that this effect was driven by auditory deprivation, and not language experience (Cardin, 2013). The absence of an early sensory processing response in Leonard's study (Leonard et al., 2012), also suggests a lack of involvement of the primary auditory cortex in visual processing.

In summary, in deaf individuals, secondary auditory areas can be activated by complex visual stimuli, independently of their linguistic content. Thus crossmodal reorganisation in these regions seems to be a result of deafness itself, rather than a result of compensatory strategies employed by the deaf person in order to access language. Therefore, these findings speak to the importance of early implantation, but not in favour of avoiding visual communication. There is no compelling evidence that visual processing is capable of colonising A1. Language-processing regions in left superior temporal cortex maintain their amodal language function, and therefore are responsive to sign language stimulation. There is no evidence visual language causes maladaptive plasticity in auditory cortex.

The crossmodal reorganisation of somatosensation in congenital deafness has also been investigated (Levanen et al., 1998; Levanen and Hamdorf, 2001; Karns et al., 2012). Somatosensation has been proposed to be important in the sensory experience of deaf people, specifically in substituting for auditory input, since skin receptors in the ear or bone conduction with hearing aids can deliver speech-related signals to the deaf ear (Auer et al., 2007). To investigate whether this causes cortical reorganisation in deaf people, the hands of hearing and early deafened participants were touched whilst they underwent fMRI (Auer et al., 2007). All the deaf participants had extensive experience with hearing aids. Somatosensory stimuli with structure similar to speech activated auditory cortex for both groups; however, activation also occurred for the unstructured tactile stimuli, and was greater and more widespread throughout auditory cortex (including putative primary auditory areas) for the deaf group (Auer et al., 2007). Thus there is increased somatosensory representation in deaf people in auditory cortex, but there is no evidence this is linked to speech processing (Auer et al., 2007).

These results are in agreement with studies in animals, which posit an anatomically feasible model of cortical reorganisation after auditory deprivation. In deafened ferrets (perinatal ototoxic lesion), single cell recordings have been made in the auditory core, which demonstrated that the majority (80%) of cells were responsive to somatosensory input (Meredith and Allman, 2012). However, anatomical tracer injections into auditory cortex displayed the same profile of connectivity as that observed in hearing ferrets (Meredith and Allman, 2012). The authors argue that neither latent nor new projections to the cortex are responsible for crossmodal plasticity, but instead that this is evidence to support the ‘brainstem theory of reorganisation’ (Meredith and Allman, 2012). In this theory, the somatosensory inputs which are found in typically developing auditory brainstem at several nodes, as well as dorsal cochlear nucleus and inferior colliculus, are responsible for crossmodal plasticity both in sub-cortical structures and throughout the cortex (Meredith and Allman, 2012).

It should be noted that evidence from animal studies also suggests a degree of visual, as well as somatosensory, crossmodal plasticity driven by auditory deprivation in core auditory areas. After auditory deprivation, visual crossmodal plasticity has been shown in mice core auditory areas A1 and AAF (Hunt et al., 2006), and somatosensory crossmodal plasticity also in A1 and AAF in mice and ferrets (Hunt et al., 2006; Meredith and Allman, 2012). In congenitally deaf cats, no evidence was found for visual or somatosensory crossmodal plasticity in A1 (Kral et al., 2003). However, in AAF, neurons do show responses to visual and, more strongly, somatosensory stimulation (Meredith and Lomber, 2011). In deaf humans it is less obvious if there is also visual crossmodal plasticity in these regions due to problems with cross-species comparisons, and because the exact composition of the core auditory cortex and its functional organisation is still a matter of controversy.

The extent of somatosensory crossmodal plasticity in primary auditory regions underscores the fact that crossmodal reorganisation of auditory cortex in deafness is neither the result of, nor exacerbated by, the use of a visual language. Again, this research speaks to the importance of early implantation, but not avoiding visual language.

## 6 What are the consequences of depriving a child of early language?

While crossmodal reorganisation in auditory cortex occurs inevitably as a result of early deafness, we have pointed out that this may not be reliably due to the influence of visual language. Early language exposure is essential for the development of language processing circuitry in the brain. This is because core language regions appear agnostic to the modality of language input, and yet sensitive to delay in language exposure and acquisition.

The critical period hypothesis of language development argues that children who fail to learn language before the end of childhood will not reach a ‘native-like’ level of mastery with the language, with full command of syntax, phonology and verbal working memory (Lenneberg, 1967). Apart from severe cases of abuse and neglect, hearing children are exposed to sufficient language in order to develop language mastery. However, for the 90–95% of deaf children who are born to hearing parents, language learning can be less robust, as they are unable to fully access the language of their care givers. Signed and spoken language tests have shown that in deaf people with insecure first language learning, syntactic processing remains rudimentary, and morphological and phonological skills are relatively poor compared to deaf native signers (Mayberry et al., 2002; Mayberry et al., 2011; Cormier et al., 2012) (see Box 1). Deaf children who used either speech reading or sign language from early in infancy performed comparably to hearing bi-lingual children in a test of English proficiency, outperforming deaf children (late L1 learners of sign language) who were unable to access the spoken language of their care giver (Mayberry, 2002). A MEG study of 2 deaf adolescents who had inconsistent language input until 14 years of age showed that viewing recently acquired signed words activated a network of regions including right superior parietal cortex, anterior occipital cortex and dorsolateral prefrontal cortex, and not the classic perisylvian network recruited for language processing, providing additional evidence that early language deprivation is likely to lead to aberrant cortical circuitry for language processing (Ferjan Ramirez et al., 2013). Overall, these results suggest that early language exposure, and not language modality, is the critical factor to secure language development.

Language deprivation (and late first language learning) can have effects beyond those pertaining solely to linguistic efficiency. Where communication with others is impaired (as is common for deaf children of hearing parents) a degree of social isolation can follow. Although there are no studies directly addressing this in deaf individuals, a mouse model has shown a sensitive period for myelination and frontal lobe development that is affected by social isolation (Makinodan et al., 2012). The effects of such reduced myelination as a result of social isolation are long term (Makinodan et al., 2009).

Even though late first (sign) language learners may achieve a high level of proficiency and fluency, there is evidence that atypical structural and functional circuitry for language processing persists in adulthood. In testing syntactic and phonological perception skill for sign language, activation varied as a function of when 22 deaf participants learned sign language as their first language (Mayberry et al., 2011). Those who learned sign language from birth activated classic perisylvian language regions, whereas late learners of sign language as an L1 demonstrated more posterior visual activation, which the authors argued was consistent with processing sign language at a shallower level (Mayberry et al., 2011). Deaf native signers have the typical electrophysiological signature upon encountering a syntactic anomaly within an utterance (Capek et al., 2009). This has also been shown to be the case when deaf native signers were tested in their second language (written German) (Skotara et al., 2011). However, deaf late L1 learners of sign language did not show this effect, even when their levels of proficiency matched those of the deaf native signing participants (Skotara et al., 2011). These neural findings accord with psycholinguistic findings of poor syntactic and morphological skill in late L1 learners of SL, proving that early language input is a prerequisite for typical language development (see Box 1). Delayed sign language acquisition has also been linked to decreased grey matter volume in visual cortex, relative to both deaf early learners of sign language as an L1 and hearing controls, suggesting the effects of language deprivation are not restricted to language processing circuitry (Penicaud et al., 2012).

The possibility that insecure first language acquisition may contribute to poor CI outcomes, and to the abnormal patterns of activation which extend beyond temporal regions when attempting language tasks, has not been considered or tested empirically. Instead of subverting speech processing regions, an early and well-established visual language may contribute to CI efficacy, both through providing language to multimodal language circuits, and in giving the child a gateway to understanding the auditory signal.

## 7 Conclusion

In this review we set out to examine the relationship between visual language and cochlear implant success. Animal models of cochlear implant have greatly enhanced our understanding of the dystrophic changes which occur when auditory cortex fails to develop typically due to the absence of auditory input. However, we argue that animal models are insufficient to characterise the cochlear implant sensitive period, as, in addition to the development of the auditory system, this is also influenced by language sensitive periods. Visual processing is argued to cause functional decoupling of auditory cortex, such that the patterned firing required to establish interconnected circuits between higher and lower auditory cortices cannot take place, as higher auditory cortex is reorganised into the visual processing stream. Theories regarding visual takeover of auditory cortex have led clinicians and researchers to advocate preventing the child from experiencing visual language prior to implant. We do not challenge the notion that visual takeover of auditory cortex is apparent in deaf people, just the assumption that this is driven by visual language. Sign language skills, measured appropriately in terms of sensitivity to the syntactic and morphological features, characteristic of language mastery in native language users, have never been measured in relation to CI outcomes. Instead, researchers have been content to identify deaf participants’ use of sign language itself as a causal factor in poor CI outcome. Experience with speech reading has also been implicated in poor outcomes. However, here, as with sign language, experience with visual language in deafness tends to be correlated with duration of deafness, age of first language acquisition and language proficiency. When these factors are controlled for, exposure to visual language cannot be linked to poor CI outcome. Instead, there are numerous studies which suggest the contrary: that proficiency with speech reading is linked to better CI outcome. Imaging studies show that visual activation during speech reception over time following CI becomes more specific, suggesting that in the CI brain, auditory and visual information mutually reinforce one another. Furthermore, even though visual motion and somatosensation can have an enhanced representation in auditory cortex following crossmodal reorganisation, these differences have not been conclusively linked to functional differences between deaf people, such as the use of a visual language. Finally, we stress the consequences of failure of early language acquisition. Evidence from deaf people who have failed to develop spoken language in an oral environment suggests that when sign language is learnt later in life, it will never display the typical neural circuitry of natively learnt languages. What do these arguments mean for the clinical management of CI in prelingual deafness? Far from shielding the developing infant from visual communication through seen speech and sign, the deaf child awaiting CI needs language input to enable effective cognitive development to proceed. The early years, including the first year of life, are crucial for the development of language, not just heard speech. Post-implant, while auditory rehabilitation is clearly necessary to enable effective functioning of the CI, we find no compelling evidence that visual language is detrimental to CI success. On the contrary, successful cochlear implantation appears to depend upon audiovisual integration skills. Early cochlear implantation is an astonishing breakthrough in delivering functional hearing to the child born deaf; however, language skills and cognitive development should not be overlooked when considering the efficacy of CI.

## Figures and Tables

**Table 1:** What is visual language? For the purposes of this review article, we define visual language as language, or a language derivate, perceived in the visual modality.

Visual language	Explanation	Notes
Speech Reading (Lip Reading)	Deducing the content of speech from viewing orofacial gestures.	Information about articulation is partially visible: the tongue is the major articulator and is often hidden within the mouth. Despite this, excellent speechreading can be achieved by some people (Campbell, 2008).

Visual Phonics (Cued Speech)	Specific, consistent manual actions are used simultaneously with seen speech to provide disambiguating phonological information.	This has been designed to support spoken language between hearing caregivers and deaf children (Narr and Cawthon, 2010).

Sign Supported Speech (SSS)	Speechreading accompanied by manual signs. Unlike sign languages, the signs are not part of any formalised grammatical system. Unlike Cued Speech, the signs do not provide discrete phonological information. The signs follow the order of the spoken language, are typically used to indicate lexical items, and can be considered as a means of providing additional semantic information to the perceiver.	SSS is used to communicate with people who may be deaf or language-impaired or who have problems with speech articulation. Although developed from distinct theoretical bases, Simultaneous Communication (SC) and Total Communication (TC) can be considered forms of SSS, since they afford a means for hearing and deaf people to communicate using a mixture of speech and signs. TC may be implicated especially in language rehabilitation in CI (Knoors and Marschark, 2012).

Sign Language	Sign languages are the natural languages of deaf communities. Hands, arms, upper torso, and face actions (including mouth actions) are all used in sign languages. Approximately 200 sign languages have been identified, reflecting spontaneous development within deaf communities. They have their own grammars, distinct from the spoken language of the surrounding community	Sign languages, unlike the other forms of visual communication (see above) demonstrate key linguistic universals in the domains of phonology, semantics and syntax. When acquired as a first language, sign language and spoken languages are processed in similar brain regions (see (MacSweeney et al., 2008a) for review).

## Box 1 Sign Language–Linguistic Evidence for a Sensitive Period in Acquisition

Sign languages are a class of natural human language which are produced by movements of the hands and arms and perceived visually (gestural-visual modality). Sign languages are not derived from or related to the spoken languages of the surrounding hearing population and arise spontaneously wherever deaf communities come into existence (Sutton-Spence and Woll, 1999). In families where parents are deaf and use sign language, children acquire a sign language as a native language. Deaf children born to hearing parents have in the past learned sign language in childhood when they have attended schools for the deaf with deaf children who sign, or more recently through early intervention programmes. All intervention programmes using sign language have as their goal bilingualism in a sign language and a spoken/written language (Mitchiner et al., 2012). Most studies distinguish between children exposed to sign language by the age of 4 (these may be native or early signers - ES) from those exposed to SL following failure to acquire a spoken language as a first language (SL as a late L1) and those who learn an SL following acquisition of a spoken language (SL as an L2).

The course of language acquisition is remarkably similar for children developing a signed or spoken language, despite the modality differences (Schick, 2003). However, studies have also shown consistent differences between native signers and late L1 learners, providing evidence for the existence of a sensitive or critical period for sign language acquisition, paralleling that for spoken language. Deaf children may only be exposed to a sign language after failing to acquire a spoken language and this late exposure to an accessible first language generally results in long-term language deficits. Studies have used a wide variety of tasks comparing deaf adults who acquire a sign language in infancy with late L1 learners. Differences have been reported in use of verb morphology (Newport, 1990), sentence shadowing and recall (Mayberry and Eichen, 1991, Mayberry, 1993), grammaticality judgments (Boudreault and Mayberry, 2000; Cormier et al., 2012), and language processing (Emmorey and Corina, 1990, Emmorey et al., 1995). For example, Cormier et al. (2012) found that the accuracy of grammaticality judgements for sentences of British Sign Language decreases as age of sign language acquisition increases for the late L1 group.

Language comprehension is also affected by delayed exposure. Late L1 learners take longer and need more visual information than native signers to identify signs (Emmorey and Corina, 1990). English grammatical abilities of deaf adults who either did or did not have linguistic experience (spoken or signed) during early childhood were investigated with grammaticality judgement and sentence-to-picture matching tasks (Mayberry and Lock, 2003). Deaf adults who were late L1 learners performed poorly.
